# Gene-by-Sex Interactions: Genome-Wide Association Study Reveals Five SNPs Associated with Obesity and Overweight in a Male Population

**DOI:** 10.3390/genes14040799

**Published:** 2023-03-26

**Authors:** Maria-Anna Kyrgiafini, Theologia Sarafidou, Themistoklis Giannoulis, Alexia Chatziparasidou, Nikolaos Christoforidis, Zissis Mamuris

**Affiliations:** 1 Laboratory of Genetics, Comparative and Evolutionary Biology, Department of Biochemistry and Biotechnology, University of Thessaly, Viopolis, Mezourlo, 41500 Larissa, Greece; 2 Laboratory of Biology, Genetics and Bioinformatics, Department of Animal Sciences, University of Thessaly, Gaiopolis, 41336 Larissa, Greece; 3 Embryolab IVF Unit, St. 173-175 Ethnikis Antistaseos, Kalamaria, 55134 Thessaloniki, Greece

**Keywords:** genome-wide association study (GWAS), obesity, overweight, body mass index, sex

## Abstract

Obesity is a chronic health problem associated with severe complications and with an increasing prevalence in the Western world. Body-fat composition and distribution are closely associated with obesity, but the human body’s composition is a sexually dimorphic trait, as differences between the two sexes are evident even from fetal life. The effect of sex hormones contributes to this phenomenon. However, studies investigating gene-by-sex interactions for obesity are limited. Therefore, the aim of the present study was to identify single-nucleotide polymorphisms (SNPs) associated with obesity and overweight in a male population. A genome-wide association study (GWAS) that included 104 control, 125 overweight, and 61 obese subjects revealed four SNPs associated with overweight (rs7818910, rs7863750, rs1554116, and rs7500401) and one SNP (rs114252547) associated with obesity in males. An in silico functional annotation was subsequently used to further investigate their role. Most of the SNPs were found in genes regulating energy metabolism and homeostasis, and some of them were expression quantitative trait loci (eQTL). These findings contribute to the understanding of the molecular mechanisms underlying obesity-related traits, especially in males, and pave the road for future research toward the improvement of the diagnosis and therapy of obese individuals.

## 1. Introduction

Overweight and obesity are defined according to the World Health Organization (WHO) as excessive or abnormal fat accumulation that can lead to impaired health, and they are caused by an energy imbalance between caloric intake and expenditure [1]. Excess body weight is a global health problem, from which it is estimated that more than two billion people worldwide suffer. This number accounts for approximately 30% of the world’s population [2], and it is estimated that between 1980 and 2013, the prevalence of overweight and obesity increased by approximately 28% [3]. Affected individuals have an increased risk of comorbidities, such as cardiovascular disease, diabetes mellitus, several types of cancer, hypertension, etc. [1]. Furthermore, the treatment of obesity and obesity-related complications accounts for 21% of the total healthcare expenditure in the United States [4].

Obesity is also considered a complex trait that arises from interactions between the individual’s genetic background and environmental factors, such as diet, physical activity, pollutants, sociocultural factors, etc. [5]. For many years, research on the genetics of obesity mainly focused on genome-wide association studies (GWAS), which led to the identification of many associated variants and highlighted genes and pathways contributing to obesity development [6,7]. Despite this progress, there is an ongoing debate, as most scientists agree that many more associated single-nucleotide polymorphisms (SNPs) are to be discovered [6,7,8]. The identified variants explain only a very small proportion, around 5%, of the total variance in adiposity [9], while simulation studies propose that SNPs account for approximately 30% of the variance in body mass index (BMI) [10], the most widely used standard for classifying adiposity and somatotypes associated with overweight or obesity. Therefore, novel approaches are investigated to discover more SNPs that could provide a better understanding of the biology of obesity.

**Figure 1:**
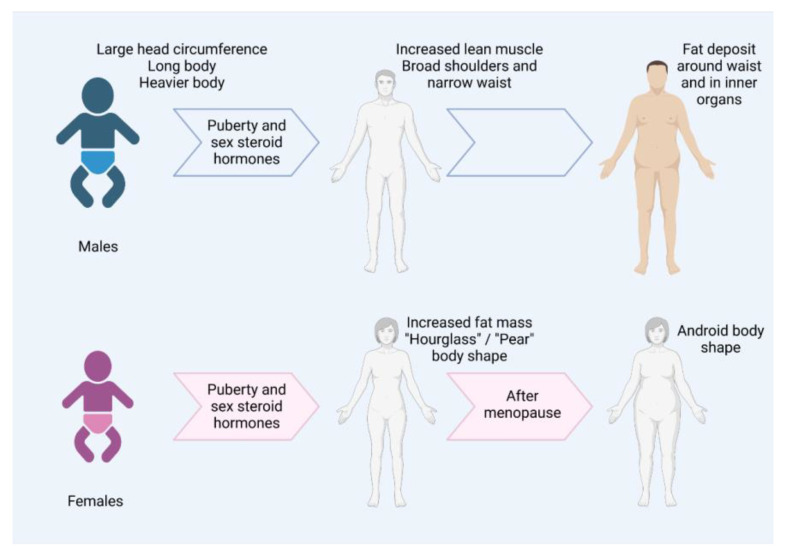
Sexual dimorphism of body shape during life according to findings of several research studies [12,13,14]. Male babies have larger head circumferences than females, as well as longer bodies, and they are heavier. During puberty, sex-steroid hormones differentiate fat distribution between the two sexes. Men have increased lean muscle and a body shape characterized by broad shoulders and narrow waists. Women have increased fat mass and “hourglass” or “pear” body shapes. Later in life, men deposit fat around the waist and in inner organs, while women tend towards an androgenous body shape. Created with Biorender.com.

Furthermore, sexual dimorphism refers to the traits that are differentiated between the two sexes, which are widespread in nature [11]. Body composition and fat distribution or body shape are sexually dimorphic traits, as differentiation is observed between males and females in both humans and nonhumans [12]. More specifically, sexual dimorphism can be observed even from birth. Males are heavier than females at birth, have longer bodies, and have larger head circumferences [12]. The sex difference, however, in fat distribution is amplified from late puberty to early adulthood [13]. During puberty, the effect of sex-steroid hormones leads to greater differentiation, as women accumulate fat mass on the thighs and hips, tending towards an “hourglass” or a “pear” body shape [12,14], while men mainly accumulate lean muscle and tend towards a body shape characterized by broad shoulders and a narrow waist [12]. Later in life, sexual dimorphism is also maintained, as women enter menopause and move towards a more androgenous body shape, which includes an increase in abdominal fat deposition [12,14]. Males also show an increase in waist circumference with age, as fat accumulates around the inner organs [12] (Figure 1). Therefore, it seems that there is an interplay between sex and body composition, and specific SNPs can be associated with overweight and/or obesity in one sex, but studies investigating the gene-by-sex interactions in overweight and obesity are limited [6,15,16].

Therefore, as most studies report SNPs associated with obesity and/or overweight in mixed samples of males and females, providing limited information about gene-by-sex interactions, we aimed to conduct a GWAS for BMI in a Greek male population to identify SNPs associated with obesity and/or overweight in males. Furthermore, we performed an in silico functional annotation to assess the biological function of candidate genes, as well as the potential roles of the SNPs identified. It should also be noted that as the available information about gene-by-sex interactions in obesity is very limited, our ultimate goal was to perform a preliminary study in order to provide a valuable reference for future research about overweight and/or obesity in males.

## 2. Materials and Methods

### 2.1. Study Participants

This study included 290 subjects, who were all Caucasian males. Semen and blood samples were collected in cooperation with the “Embryolab Fertility Clinic” (Thessaloniki, Greece) for the research program “Spermogene” (grant number Τ1ΕΔΚ-02787), aiming to study the genetic basis of male infertility. Written informed consent was given by all participants and, along with the consent form, volunteers also completed a questionnaire to obtain information about height (m), weight (kg), age, clinical and medical history, health habits, etc. All these data were used in the present study to investigate genes associated with obesity and overweight in male subjects.

At first, BMI was calculated for all the individuals as the weight (kg) divided by height squared (m^2^), after which subjects were classified as normal weight (18.5 kg/m^2^ < BMI < 24.9 kg/m^2^), overweight (25 kg/m^2^ < BMI < 29.9 kg/m^2^), and obese (BMI > 30 kg/m^2^), as presented in Table 1. More specifically, 104 men with normal weight, 125 overweight men, and 61 obese men were included in the present analysis. All individuals in the three groups were approximately the same age and had the same health habits (alcohol consumption, smoking). Individuals who received medication that could affect their BMI were excluded. Furthermore, the study was approved by the Ethics Committee of the University of Thessaly and was carried out in accordance with the guidelines of the Declaration of Helsinki.

### 2.2. DNA Extraction

The DNA was extracted from the blood and semen samples of volunteers. The DNA extraction from blood samples was performed using the PureLink Genomic DNA Mini Kit (Invitrogen, Waltham, MA, USA—Catalog number: K182002) according to the manufacturer’s instructions. For semen samples, a protocol developed by Weyrich A. [17] was used. The DNA integrity was assessed with agarose-gel electrophoresis and the amount of DNA was evaluated spectrophotometrically with a Qubit 2.0 fluorometer using the Qubit dsDNA BR Assay Kit (Invitrogen, Waltham, MA, USA—Catalog number: Q32850). Purified DNA was stored at −20 °C until use.

It should be noted that for every volunteer, DNA extracted from blood and semen samples were not combined; instead, they were used separately for subsequent analyses, although they produced the same results.

### 2.3. Genotyping

Once the preparation was completed, the DNA samples were shipped to the Erasmus MC Human Genomics Facility (HuGe-F, University Medical Centre, Rotterdam, The Netherlands), where they were genotyped using the Illumina Infinium^®^ Global Screening Array (Illumina, San Diego, CA, USA). This chip is of high density, and it examines approximately 756400 SNPs across the human genome.

### 2.4. Quality Control (QC)

Genotyping data were obtained in .ped and .map files and, subsequently, PLINK software v1.07 [18] was used for the QC procedures and statistical analyses that followed. 

Extensive quality control was performed, and strict criteria were used to generate reliable results and avoid errors and false-positive results that may have arisen due to poor quality of DNA samples, poor DNA hybridization, contamination of samples, etc. Therefore, for quality control, at first, we removed SNPs with missing genotyping data on more than 10% of the samples, as well as samples with more than 10% missing genotypes. Furthermore, we discarded SNPs with a minor-allele frequency (MAF) of less than 3% and checked the Hardy–Weinberg equilibrium to exclude SNPs that deviate from it in controls (*p*-value < 0.001). We also excluded additional samples based on heterozygosity and relatedness tests, and SNPs were pruned to remove these in linkage disequilibrium.

### 2.5. Association Analysis and In Silico Functional Annotation

The PLINK software v1.07 [18] was used to perform association analyses between SNP genotypes and phenotypes of interest based on standard linear regression. Obesity and overweight were considered binary traits according to the categories described above (normal weight, overweight, obese) and defined by the BMI value. More specifically, two association analyses were performed. In the first, the control group consisted of individuals with normal BMI (*n* = 104), and the case group consisted of overweight individuals (*n* = 125). In the second association analysis, the control group again included individuals with normal BMI (*n* = 104), and the case group included obese individuals (*n* = 61). For all the association analyses, the chi-square test was used and the significance threshold for SNP–trait associations was a *p-*value < 10^−5^. Manhattan plots and quantile–quantile (Q–Q) plots were generated with the *qqman* package [19]. The Manhattan plots were used to visualize the association analyses, whereas Q–Q plots showed the observed and the expected distribution of the statistical tests to assess potential systematic biases. 

Finally, to investigate the potential regulatory role of these loci, the SNPs identified as significant for every association study were annotated to detect candidate genes associated with obesity and overweight in males. Gene annotation was based on data provided by Ensembl [20] and using the GRCh38 reference genome. In addition, SNPnexus [21], GTex (Genotype-Tissue Expression Project) [22], 1000 Genomes [23], Polymorphism Phenotyping v2 (PolyPhen2) [24], Sorting Intolerant From Tolerant (SIFT) [25], RegulomeDB [26], and 3DSNP [27] databases were used to obtain more biological information about regulatory elements, population genetics, associations with other phenotypes and diseases, expression quantitative trait loci (eQTLs), etc. Among these, RegulomeDB [26] is a database classifying SNPs according to the presence or absence of functional elements, such as protein binding, motifs, chromatin structure, eQTLs, histone modifications, etc. More specifically, each SNP was assigned a rank ranging from 1 to 7, with lower values representing SNPs with a higher probability of having a regulatory function. The 3DSNP [27] is another integrated database that provides information on 3D-interacting genes, enhancer states, promoter states, transcription factor binding sites, altered sequence motifs, and conservation, to calculate a functional score for every SNP. Higher scores indicate a higher likelihood of SNP functionality. Finally, the miRNASNP-v3 database [28] was used to investigate whether the significant SNPs created or destroyed miRNA binding sites, affecting the miRNA binding affinity with target mRNAs, indicating another regulatory role of SNPs.

## 3. Results

To identify SNPs associated with overweight, we genotyped 104 controls (normal BMI) and 125 cases (overweight, 25 kg/m^2^ < BMI < 29.9 kg/m^2^) for approximately 756,000 SNPs. After QC with strict criteria, including, among others, the removal of samples with missing genotypes, SNPs with very low MAF, the relatedness test, etc., 94 controls, 118 cases, and 362,108 SNPs remained for the association analysis with the chi-square test. For the GWAS on obesity, we used the same control group (104 individuals with normal BMI) and 61 cases (obese, BMI > 30 kg/m^2^). After QC, 94 controls, 57 cases, and 356,016 SNPs remained for the subsequent analyses.

**Figure 2:**
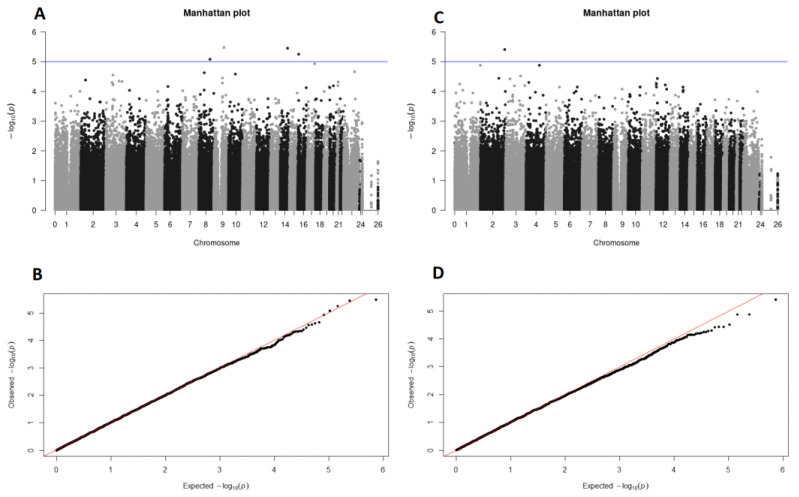
(**A**,**C**) Manhattan plots of genome-wide-association study data show SNPs associated with overweight (**A**) and obesity (**C**). The X axis represents the genomic coordinates of SNPs on respective chromosomes, while the Y axis represents the significance level on a −log_10_ scale. The suggestive-significance threshold is indicated by the blue horizontal line (*p*-value = 10^−5^). Four SNPs were found to be associated with overweight (**A**) and one SNP with obesity (**C**); (**B**,**D**) quantile–quantile plots of the association analysis between men with normal BMI and overweight men (**B**) and between men with normal BMI and obese men (**D**). The X axis represents the expected significance level on a −log_10_ scale, while the Y axis represents the observed significance level on the same scale.

The first GWAS resulted in four SNPs that surpassed the suggestive-significance threshold (*p-*value < 10^−5^) and were found to be associated with overweight, while only one SNP passed the threshold in the GWAS for obesity. The SNPs found to be associated with overweight were found on chromosomes 8, 9, 14, and 16, whereas the SNP associated with obesity was mapped on chromosome 2. The Manhattan plot and the Q–Q plots showing the distribution of observed and expected *p*-values for both association analyses are presented in Figure 2.

As also shown in Table 2, two of the significant SNPs identified in the first GWAS were associated with a higher risk of overweight (rs7818910, OR = 2.81; rs1554116, OR = 2.529), while the other two SNPs were associated with a lower risk (rs7863750, OR = 0.2582; rs7500401, OR = 0.3421). The SNP associated with obesity also had a high odds ratio (15.18), indicating a strong association of the variant with obesity.

Next, to perform functional annotation of all the statistically significant SNPs and investigate their potential role in overweight and obesity, several databases were used, as explained previously. At first, population genetics data and information about allele frequencies in five populations were obtained using SNPnexus [21] and the 1000 Genomes [23] databases. As shown in Table 3, one of the SNPs associated with overweight (rs7863750) had a very low minor-allele frequency, below 0.05, whereas the minor-allele frequency of the SNP associated with obesity was 0.0157.

To further investigate the potential roles of the SNPs identified, we used several other databases for their functional characterization, as described above (Table 4). For overweight, two of the SNPs were found in intergenic regions and one in an intronic region. More specifically, rs1554116 was found in an intronic region of *KCNK13* that encodes for a two-pore-domain potassium channel (K_2p_ channels). The K_2p_ channels may play an important role in thermogenesis and metabolism homeostasis, according to previous research [29,30,31]. The rs7863750 is the only significant SNP mapped on a coding region, and for one gene transcript, it is also considered a non-synonymous mutation. According to SIFT [25] (tolerated, score: 0.640) and PolyPhen2 (benign, score: 0.00) [24] scores, this variant does not affect the protein’s function or structure. However, the same SNP is also an expression quantitative trait locus (eQTL) that affects the expression of *MFSD14B* in the adipose tissue, according to GTex [22]. More specifically, this SNP increases the expression of *MFSD14B*. Furthermore, none of the SNPs identified had a score indicating high functionality, according to RegulomeDB [26] and/or 3DSNP [27]. The SNP found to be associated with the obesity phenotype is an intronic variant mapped on the *DGKD* gene and it is not an eQTL. Furthermore, this SNP has a RegulomeDB score of 0.13 and its RegulomeDB rank is 5. In addition, it has a 3DSNP score of 10. Finally, the significant SNPs were not found to disrupt regions that interact with miRNAs, according to the miRNASNP-v3 database [28].

## 4. Discussion

In the present study, to systematically investigate the gene-by-sex interactions observed in obesity and overweight, we used GWAS to identify SNPs associated with these traits in a male population, after which we tried to investigate their potential role, or the role of candidate genes, in the complex mechanisms involved in body mass composition and body shape. Our genome-wide search identified four SNPs associated with overweight and one SNP associated with obesity in Greek males.

More specifically, the first genome-wide association study performed here included 104 males with normal BMI (controls) and 125 males with BMI classifying them as overweight (cases). After QC, 94 controls and 118 cases remained for the subsequent analysis. The SNPs that were significantly (*p-*value < 10^−5^) associated with overweight in this study were rs7818910, rs7863750, rs1554116, and rs7500401. Notably, none of these SNPs were found to be associated with BMI or other obesity-related traits in previous genome-wide association studies. Half of these SNPs are risk alleles (rs7818910, rs1554116), while the others are protective alleles (rs7863750, rs7500401), according to the OR. Except for rs7863750, all the SNPs are found in intergenic or intronic regions. 

The rs1554116 gene, found in an intronic region of *KCNK13*, may be a good candidate for future research as it plays an important role in thermogenesis and obesity, according to previous research. It encodes for a two-pore-domain potassium channel, and these channels are highly expressed in brown and beige adipose tissue [29,30]. More interestingly, Yi Chen et al. (2017) [31] proved that another member of the gene family, *KCNK3*, acts as a negative regulator of thermogenesis and energy expenditure by performing experiments on mice. It seems that the potassium channel limits calcium influx, as it antagonizes norepinephrine-induced membrane depolarization by increasing potassium efflux in brown adipocytes. Furthermore, the adipose-specific knockout mice that the scientists used in their study had increased energy expenditure and were resistant to hypothermia and obesity [31]. It should also be noted that the *KCNK3* locus was identified as associated with BMI in another GWAS study, on humans [32]. These findings suggest that K_2p_ channels may play a role in the regulation of energy metabolism.

Although rs1554116 is an intronic variant of *KCNK13*, and intronic variants do not have a direct effect on protein production, they can still affect the functionality of a gene. Intronic variants that cause abnormal splicing changes by destroying existing splicing motifs or by creating new ones are an important class of pathogenic variant, as they can affect the protein’s function, and they account for 15–60% of human disease variants [33]. Interestingly, however, some intronic variants, despite not being present at splice junctions, are still considered functional, and they can change the splicing phenotype because they are located in an intron splice enhancer or branchpoint site, or because they are activators of cryptic splice sites [34,35]. Cooper (2010) [36] summarized several of these functional variants, many of which are located at least ~30 base pairs (bp) from the nearest splice site. These SNPs are usually overlooked, but in vitro studies have shown their association with several diseases, and they have proven that they can have an impact on the transcriptional activity or the splicing efficiency of their host genes; they can also change the expression of alternative transcripts [36]. Therefore, further research is required to assess whether rs1554116 is a functional intronic variant that can affect the expression or the function of *KCNK13*, as the experimental results from previous studies suggest that changes in its expression or any other defect in the protein’s function can affect thermogenesis and energy homeostasis. As the knockout mice in the experiment performed by Chen et al. (2017) [31] were resistant to obesity due to increased energy expenditure, it is possible that rs1554116, which is considered a risk allele for obesity, can cause the increased expression of *KCNK13* and defective thermogenesis, but further research and functional experiments are required to support this assumption.

Furthermore, rs7863750 might be another promising variant that was found to be associated with a lower risk of being overweight (OR = 0.2582) in this study. According to 1000 Genomes, this SNP also has a very low MAF, below 0.05. In the present study, its frequency (0.07) was almost equal to that observed in the European population (0.08), but the frequency observed in individuals with normal BMI was extremely high (0.23). The rs7863750 is the only SNP identified in the present study to be located in a coding region. More specifically, it is considered a synonymous mutation for one transcript, but also a non-synonymous mutation for another transcript of the gene. In both cases, recent studies indicate that both synonymous and non-synonymous mutations can affect gene expression and protein function in a variety of ways [37,38,39,40]. In this case, however, as the same variant was characterized as an expression quantitative trait locus (eQTL,) it seems that rs7863750 has the potential to alter the gene’s expression. Research shows that even synonymous mutations can contribute to human diseases [37] as they can influence the stability of mRNA transcripts, e.g., by affecting the mRNA’s secondary structure, which in turn influences the abundance of mRNAs (less stable mRNAs are easily degraded) and the levels of the protein produced [37,39]. Similarly, increased levels of protein can be observed if the mutation affects the mRNA’s structure and, subsequently, the mRNA’s interactions with specific proteins [40]. The rs7863750 is associated with the increased expression of *MFSD14B* in adipose tissues, according to GTex. The MFSD14B is a membrane-bound transporter that, according to studies on mice, is found in neurons of the central nervous system and is also involved in energy homeostasis [41]. Furthermore, *Mfsd14b* is also expressed in many peripheral tissues in mice, and its highest expression is observed in the liver. In the same study, the researchers found that nutrient availability induced the up-regulation of *Mfsd14b* in primary cortex cells, while its expression was also affected by starvation [41]. Although the researchers only studied nutrient availability, previous experiments proved that in these cases, other obesity-related factors are also modified, such as hyperinsulinemia, hyperglycemia, etc. [42]. Therefore, it seems that the exact mechanism of action of *MFSD14B* and its function are not yet fully understood [43], but rs7863750 and the overexpression of *MFSD14B* may have a role in obesity, as this protein seems to be involved in the regulation of glucose metabolism and insulin signaling [41,43]. However, the role of *MFSD14B* and the variants mapped in this gene in obesity-related traits should be further investigated. Future studies may also focus on unraveling its role in adipose tissue in order to provide conclusive evidence for its association with obesity.

In addition, rs7500401 may also require further research, as it is located near a lncRNA and, recently, it was suggested that lncRNAs play a regulatory role in adipogenesis and obesity [44,45,46], although the available information is still limited. 

For the second genome-wide association study, 104 control samples with normal BMI and 61 cases (obese, BMI > 30 kg/m^2^) were used. After QC, 94 controls and 57 cases remained. The analysis showed that only one intronic SNP (rs114252547) of *DGKD* was associated with obesity in men (*p-*value < 10^−5^). Interestingly, this specific variant has a high odds ratio (15.18). More specifically, the frequency in Europe is estimated at 0.03, but its frequency in the cases in this study was 0.14, almost five times higher. Furthermore, although the SNP has never been associated with obesity or obesity-related traits in the past, the gene in which it is found is involved in metabolic regulation. The *DGKD* encodes for a diacylglycerol (DAG) kinase (DGK), which plays a unique role in lipid metabolism, since it acts both as an intermediate product of triglyceride synthesis and as a signaling molecule [47]. Elevated levels of DAG have been associated with insulin resistance and the pathogenesis of type 2 diabetes because increased DAG encourages intracellular lipid accumulation and aberrant signal transduction through the activation of protein kinase C (PKC) isoforms [48]. In this process, DGKs catalyze the phosphorylation of DAG to phosphatidic acid (PA), attenuating DAG signaling [49]. Based on this function, scientists assumed that DGKs can play a role in fat-deposition regulation; therefore, in an attempt to unravel the role of, Jiang et al. (2016) [50] demonstrated that its reduction affects AMPK signaling and lipid metabolism by altering DAG content. Previous experiments also showed that DGKδ^+/−^ mice were obese and developed skeletal-muscle insulin resistance with age [51]. All these findings suggest that a reduction in DGKδ can lead to obesity through increases in insulin resistance and metabolic inflexibility. Therefore, the SNP identified in this study as associated with obesity in males and found in *DGKD* may be a promising candidate for future research. 

**Figure 3:**
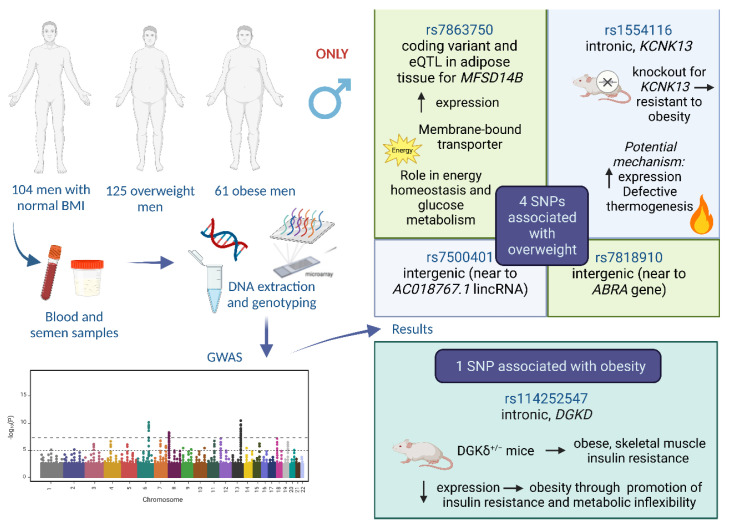
Summary of the methodology and the results of the present study. In brief, the genome-wide association study (GWAS) that included 104 control, 125 overweight, and 61 obese subjects revealed four SNPs associated with overweight (rs7818910, rs7863750, rs1554116, and rs7500401) and one SNP (rs114252547) associated with obesity in males. The potential role of the identified SNPs in overweight and obesity is also presented based on previous studies and research [29,30,31,32,41,42,47,48,49,50]. The figure was created with Biorender.com.

In summary, the research methodology, as well as the results of the present study and the potential role of the SNPs identified, are presented in Figure 3.

This study has some strengths and limitations. First, the main strength of the study is the fact that it is one of the few to investigate gene-by-sex interactions in obesity and body mass. Although sexual dimorphism plays a key role in fat distribution [15], only a few studies about obesity are designed to explicitly focus on females or males only. Thus, this study, conducted exclusively on males, can provide novel insights into human physiology and the development of obesity, as well as biological clues to aid in disease prevention and treatment. Second, another strength is the stringent quality-control criteria used. The SNPs and samples that could have affected the quality of our results and led to false-positive or false-negative results were excluded through the rigorous quality control, which included several steps, as described above. Finally, another strength is the genetic homogeneity of our sample. All the volunteers belonged to the Greek population, according to the questionnaire that they completed before their enrolment in the present study. Genetic heterogeneity can affect the ability to detect associations between genotypes and phenotypes of interest [52] and, especially in obesity, ethnicity differences have been reported due to genetic background [53,54]. However, some limitations should be considered. The major limitation of the present study is the small sample size that was used, as only 125 overweight and 61 obese individuals were included in the GWAS. After QC, the number of individuals included was even lower, indicating that this study was underpowered. Therefore, more research and large-scale genetic studies are required to assess the role of the SNPs identified here in obesity and overweight. Furthermore, in the present study, we did not identify SNPs associated with obesity or overweight in males at the genome-wide significance level (*p*-value < 5 × 10^−8^). Finally, it should be noted that this analysis was performed on males, and we did not perform the same analysis on females. As our sample was composed exclusively of males, it is possible that the SNPs identified here are associated with obesity and overweight in males and thus, have a male-specific effect. However, we cannot draw definitive conclusions about this sex-specific effect, as we did not study a female population. Therefore, further research and, potentially, another genome-wide association study focusing on the female population are suggested for future study.

Finally, this is a preliminary study aiming at the investigation of SNPs that are potentially associated with overweight and/or obesity in males, and it does not provide conclusive evidence on specific SNPs and their impact. The results presented here should be interpreted with caution and validated in larger, independent cohorts, as our study had several limitations. However, smaller studies such as this are still of importance. Especially in cases in which the available information is limited, as in gene-by-sex interactions in obesity and/or overweight, studies such as this can provide roadmaps for future research, enlist candidate genes and variants, confirm the roles of genes that have been studied in the past in obesity and/or overweight, and contribute to our understanding of the genetics of complex diseases and traits.

## 5. Conclusions

In conclusion, we assayed a sample collection of obese and overweight cases and controls with normal BMI, all of whom were male, and performed two association analyses to identify SNPs associated with overweight and obesity. We identified five associated SNPs with *p-*values < 10^−5^ that may contribute to obesity and/or overweight in a sex-specific manner. It should also be noted that although most of the genes in which the significant SNPs were found play a role in energy metabolism and homeostasis, the identified SNPs were not previously reported in relevant studies. This indicates that the heritability of obesity-related traits is not attributable to only a few genetic variants. Therefore, the findings presented in the present study can help guide future research on the characterization of the genetic risks of obesity-related traits and on the unraveling of the molecular mechanisms of obesity and/or overweight, helping to better prevent and treat these conditions.

## Figures and Tables

**Table 1:** Male subjects included in the study categorized according to their BMI (m/kg2).

	Normal (*n* = 104)	Overweight (*n* = 125)	Obese (*n* = 61)
**BMI (m/kg^2^)** **mean, SD**	19.1–24.9 23.14 (1.48)	25–29.9 27.05 (1.41)	30.1–47.6 33.12 (2.94)
**Age (years)** **mean, SD**	19–52 32.3 (7.19)	22–49 36.7 (5.37)	26–53 39.34 (5.65)
**Smoking (Yes/No)**	No, *n* = 59 (56.7%) Yes, *n* = 45 (43.3%)	No, *n* = 64 (51.2%) Yes, *n* = 61 (48.8%)	No, *n* = 39 (63.9%) Yes, *n* = 22 (36.1%)
**Alcohol Consumption** **(<2 drinks/week, 2 drinks/week, >2 drinks/week)**	<2 drinks/week, *n* = 64 (61.6%) 2 drinks/week, *n* = 20 (19.2%) >2 drinks/week, *n* = 20 (19.2%)	<2 drinks/week, *n* = 67 (53.6%) 2 drinks/week, *n* = 34 (27.2%) >2 drinks/week, *n* = 24 (19.2%)	<2 drinks/week, *n* = 41 (67.2%) 2 drinks/week, *n* = 13 (21.3%) >2 drinks/week, *n* = 7 (11.5%)

**Table 2:** Summary of the association results for overweight and obesity, significant SNPs, and their genomic position; Chr, chromosome; Ref/Alt, reference/altered; OR, odds ratio.

CHR	SNP	Position	Ref/Alt Allele	Frequency Cases	Frequency Controls	*p-*Value	OR
**SNPs associated with overweight**							
8	rs7818910	107979407	A/C	0.3686	0.172	8.344 × 10^−6^	2.81
9	rs7863750	97221463	G/A	0.07203	0.2312	3.356 × 10^−6^	0.2582
14	rs1554116	90570376	C/A	0.572	0.3457	3.534 × 10^−6^	2.529
16	rs7500401	8495998	C/T	0.1441	0.3298	5.64 × 10^−6^	0.3421
**SNP associated with obesity**							
2	rs114252547	234311271	T/G	0.1404	0.01064	3.921 × 10^−6^	15.18

**Table 3:** Allele frequencies in five populations for the SNPs found to be associated with overweight and obesity; Ref/Alt, reference/altered; MAF, minor-allele frequency; EAS, East Asian; AMR, American; AFR, African; EUR, European; SAS, South Asian.

Variant ID	Ref/Alt Allele	MAF	EAS	AMR	AFR	EUR	SAS
**SNPs associated with overweight**							
rs7818910	A/C	0.48	0.378	0.653	0.272	0.719	0.522
rs7863750	G/A	0.04	0.001	0.032	0.006	0.084	0.071
rs1554116	C/A	0.47	0.535	0.491	0.399	0.528	0.424
rs7500401	C/T	0.36	0.489	0.429	0.194	0.259	0.510
**SNP associated with obesity**							
rs114252547	T/G	0.0157748	0	0.0159	0.0008	0.0328	0.0348

**Table 4:** Annotation and functional characterization of the SNPs found to be associated with overweight and obesity according to GTex portal [22], RegulomeDB [26], and 3DSNP [27].

SNP	Closest Gene	SNP–Gene Distance	Annotation	eQTL	RegulomeDB Score	3DSNP Score
**SNPs associated with overweight**						
rs7818910	*ABRA*	196 kb	Intergenic	No	Rank = 7, Score = 0.18412	1.69
rs7863750	*MFSD14B*	0 kb	Coding	Yes (Adipose Tissue—Visceral and Subcutaneous)	Rank = 5, Score = 0.13454	0
rs1554116	*KCNK13*	0 kb	Intronic	No	Rank = 4, Score = 0.60906	1.59
rs7500401	*AC018767.1* (lincRNA)	70 kb	Intergenic	No	Rank = 5, Score = 0.13454	0.79
**SNP associated with obesity**						
rs114252547	*DGKD*	0 kb	Intronic	No	Rank = 5, Score = 0.13454	10.49

## Data Availability

The data presented in this study are available on request from the corresponding author.
